# Association of blood urea nitrogen to high-density lipoprotein ratio with all-cause and cardiovascular mortality in individuals with mildly reduced eGFR

**DOI:** 10.1016/j.clinsp.2026.101096

**Published:** 2026-09-05

**Authors:** Shijia Jin, Yilin He, Yifan Zhang, Zhong Chen

**Affiliations:** a Department of Cardiology, Shanghai Sixth People’s Hospital Affiliated to Shanghai Jiao Tong University School of Medicine, Shanghai, China; b Department of Critical Care Unit, Shanghai Sixth People's Hospital Affiliated to Shanghai Jiao Tong University School of Medicine, Shanghai, China

**Keywords:** Blood urea nitrogen, High-density lipoprotein cholesterol, National health and nutrition examination survey (NHANES), Mildly reduced eGFR, Mortality

## Abstract

**Background:**

Patients with mild-to-moderate renal impairment face elevated cardiovascular risk, yet early detection tools remain limited. The Cardiovascular-Kidney-Metabolism (CKM) interplay underscores the need for integrated biomarkers. The Blood Urea Nitrogen (BUN) to High-Density Lipoprotein (HDL) cholesterol ratio (BUN/HDL) was therefore evaluated for early risk stratification.

**Methods:**

This cohort study included National Health and Nutrition Examination Survey (NHANES) participants (2007‒2018) with mildly reduced estimated Glomerular Filtration Rate ([eGFR] < 90 mL/min/1.73 m^2^). Associations of BUN/HDL with all-cause and cardiovascular mortality was examined using survey-weighted multivariable Cox regression. The Boruta algorithm with Adaptive Best Subset Selection (ABESS) identified optimal predictor sets. Model performance was quantified by discrimination (AUC) and calibration (Brier score and calibration slope). Incremental value was assessed using net reclassification improvement (NRI) and Integrated Discrimination Improvement (IDI).

**Results:**

Elevated BUN/HDL showed a modest association with all-cause (HR = 1.050, 95% CI: 1.021–1.081) and cardiovascular mortality (HR = 1.062, 95% CI: 1.020‒1.105). Optimal cut-points were 6.41 (AUC = 0.650) for all-cause and 5.00 (AUC = 0.682) for cardiovascular mortality. Subgroup analyses suggested a stage-dependent pattern. For all-cause mortality, associations were consistent in Chronic Kidney Disease (CKD) stage G2 (HR = 1.079, p = 0.002) and G3 (HR = 1.058, p = 0.025). For cardiovascular mortality, the association was significant in CKD stage G2 (HR = 1.127, p < 0.001). This cardiovascular association was stronger in non-hypertensive individuals. Machine learning confirmed BUN/HDL as a robust predictor within NHANES dataset, with final models showing excellent discrimination (AUCs 0.820–0.837) and calibration.

**Conclusion:**

The BUN/HDL ratio demonstrates a consistent, albeit modest, association with mid-term mortality, providing preliminary support as a CKM risk indicator among individuals with mildly reduced eGFR.

## Highlights

• BUN/HDL is a novel CKM biomarker for risk stratification in mildly reduced eGFR.
• Elevated BUN/HDL predicts all-cause and CV mortality, strongest in early CKD G2.
• BUN/HDL is identified as a robust predictor for both mortalities in NHANES.

## Introduction

Renal insufficiency affects approximately 9.1% of the global population, with early stages G1‒G3 accounting for the majority (8.9%) of cases, including 5.0% in stages G1‒G2 and 3.9% in stage G3.^1^ It has been widely recognized as an independent risk factor for Atherosclerotic Cardiovascular Disease (ASCVD). Major guidelines, such as the 2021 European Society of Cardiology (ESC) guidelines, classify Chronic Kidney Disease (CKD) as a high-risk factor for ASCVD.^2^ Cardiovascular mortality risk has been reported to be two-fold and three-fold higher in patients with estimated Glomerular Filtration Rate (eGFR) levels corresponding to CKD stages 3a and 4, respectively, compared to individuals with normal kidney function.^3^ Emerging studies further confirm that mild renal impairment, even at subclinical levels, is associated with increased cardiovascular disease risk, underscoring the importance of early detection in this population.^4^ Understanding the mechanisms connecting eGFR reduction and cardiovascular disease is essential.

The Cardiovascular-Kidney-Metabolism (CKM) syndrome framework highlights the interplay among renal dysfunction, cardiovascular disease, and metabolic disturbances.^5^ Metabolic derangement initiates vascular injury and inflammation, promoting atherosclerosis and renal fibrosis.^6^ Subsequently, declining renal function activates the Renin Angiotensin Aldosterone System (RAAS) and the Sympathetic Nervous System (SNS), increasing cardiac workload and inducing ventricular remodeling.^7^^,^^8^ In turn, cardiovascular disease impairs renal function, establishing a bidirectional relationship that significantly increases the risk of cardiovascular events in kidney dysfunction patients.

Despite the acknowledged cardiovascular risk in early renal insufficiency, this condition is often asymptomatic and cardiovascular risk prediction models specifically developed for patients with mildly reduced eGFR are lacked, hindering timely detection and leading to delayed diagnoses and interventions.^9^ Moreover, given the complex cardio-renal interplay in these patients, traditional single risk factors may be insufficient. Therefore, there is a critical need for more precise, CKD-specific risk assessment tools that can enable early detection, improve risk stratification, and guide timely interventions in this high-risk population.

Blood Urea Nitrogen (BUN) is an indicator of kidney damage. Elevated BUN levels reflect impaired renal filtration capacity and may contribute directly to cardiovascular injury via mechanisms such as oxidative stress and inflammation.^10^ High-Density Lipoprotein cholesterol (HDL), a key factor in lipid metabolism, typically exerts cardio-protective effects. However, reduced HDL levels or impaired HDL function can diminish this protection.^11^ Critically, a direct pathophysiological link exists between elevated BUN and HDL dysfunction, primarily mediated by urea-driven carbamylation, a process that compromises HDL's structure and anti-atherogenic functions.^12^ These factors ‒ BUN and HDL ‒ are hypothesized to interact significantly in the context of mildly reduced eGFR, collectively disrupting the cardio-renal and metabolic balance. Based on this rationale, we introduce and validate the BUN/HDL ratio as a novel biomarker of all-cause and cardiovascular mortality in patients with mildly reduced eGFR, highlighting its potential as a clinically useful indicator of adverse outcomes in this population.

## Methods

### Study population

Data for this study were derived from the National Health and Nutrition Examination Survey (NHANES) (https://wwwn.cdc.gov/nchs/nhanes), assessing the health and nutritional status of the U.S. civilian, non-institutionalized population. To ensure national representation, NHANES uses a complex, multistage probability sampling design. The Centers for Disease Control and Prevention (CDC)-conducted survey provides publicly available data for public health research. All protocols followed NHANES ethical guidelines, including Institutional Review Board (IRB) approval and informed consent.

**Fig. 1:**
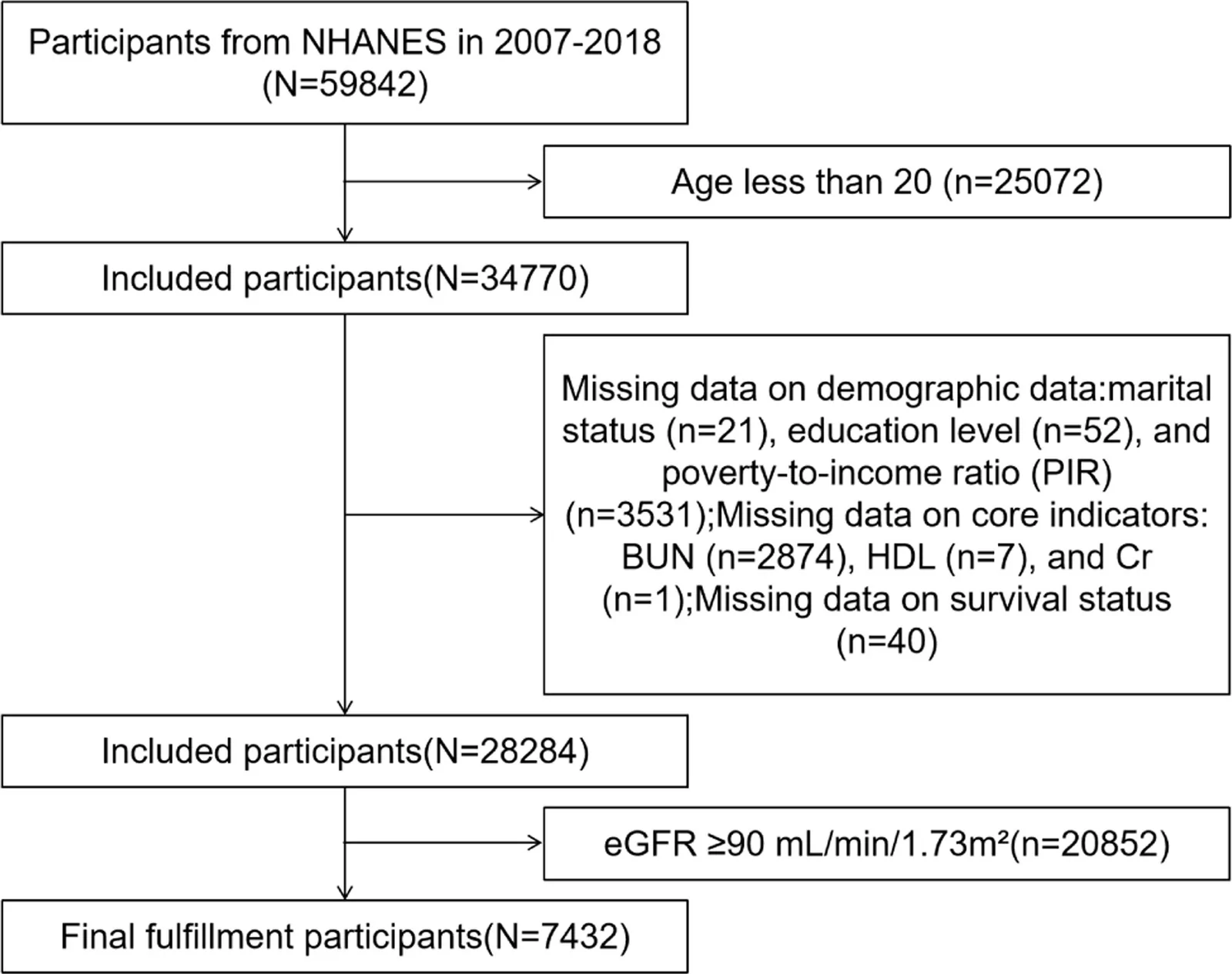
The flowchart of sample inclusion.

Data from six consecutive cycles spanning 2007 to 2018 were included, covering 59842 participants. First, individuals under the age of 20-years (n = 25072) were excluded. Secondly, participants with missing demographic data, including marital status (n = 21), education level (n = 52), and Poverty-to-Income Ratio (PIR) (n = 3531), missing core clinical indicators, including BUN (n = 2874), HDL (n = 7) and serum Creatinine (Cr) (n = 1), or missing survival status (n = 40) were excluded. Thirdly, by using the eGFR calculation formula (see Section 2.2), participants with eGFR ≥ 90 mL/min/1.73 m^2^ were excluded. The final analytic sample comprised 7432 individuals (Fig. 1).

*Calculation of eGFR and BUN/HDL ratio*

The eGFR was calculated using the 2021 Chronic Kidney Disease Epidemiology Collaboration (CKD-EPI) equation:^13^

$$\text{eGFR}=142\times \text{min}{\left(\frac{\text{Scr}}{\kappa },1\right)}^{\alpha }\times \max{\left(\frac{\text{Scr}}{\kappa },1\right)}^{\alpha }\times 0.9938^{\text{age}}\times \left(1.012\;\text{if}\;\text{female}\right)$$

where Scr is the serum creatinine level (mg/dL), κ = 0.7 for females and 0.9 for males, α = -0.241 for females and -0.302 for males and age is in years.

The BUN/HDL ratio was computed by dividing the BUN level by the HDL level, both measured in mmol/L:

$$\text{BUN}/\text{HDL}=\frac{\text{BUN}\left(\text{mmol}/L\right)}{\text{HDL}\left(\text{mmol}/L\right)}$$

### Definitions of outcomes

Mortality data was obtained from publicly accessible NHANES files, with the last recorded date being December 31, 2019. These files were then linked to the National Death Index (NDI) through a probabilistic matching process to ensure precise tracking of deaths (https://www.cdc.gov/nchs/data-linkage/mortality.htm). Death from any cause was classified as all-cause mortality. Cardiovascular mortality included deaths resulting from heart conditions (using codes I00–I09, I11, I13, or I20–I51) or cerebrovascular issues (using codes I60–I69).

### Covariates assessment

This study examined the impact of numerous covariates on the correlation of BUN/HDL with all-cause and cardiovascular mortality within mildly reduced eGFR patients. Continuous variables included age, dietary protein intake, Body Mass Index (BMI), eGFR, White Blood Cell Count (WBC), Hemoglobin (Hb), Platelet Count (PLT), Albumin (Alb), Total Cholesterol (TC), HDL, Uric Acid (UA), serum sodium, Triglycerides (TG), and the homeostasis model assessment of insulin resistance (HOMA-IR, calculated as [fasting insulin (μIU/mL) × fasting glucose (mmol/L)] / 22.5), BUN and Cr.

Categorical variables included gender (male, female), race, education level (categorized as less than high school, high school, or college and above), and marital status (married/cohabiting vs. other). Economic status was assessed using the PIR, classifying participants as either poor (PIR < 1) or not poor (PIR ≥ 1). Alcohol consumption was determined via 24-hour dietary recalls over two days; participants were classified as drinkers if they consumed alcohol on at least one recall day, versus non-drinkers. Smoking status was categorized based on self-reported questionnaires as never smoker, former smoker, or current smoker. Exercise levels were categorized into four ordered groups: sedentary (no exercise), low activity, moderate activity and high activity. Hypertension (HTN) diagnosis was based on any of these factors: systolic blood pressure measured at ≥140 mmHg, diastolic blood pressure measured at ≥ 90 mmHg during the physical examination, a self-reported physician diagnosis or current use of antihypertensive medication. Diabetes Mellitus (DM) was defined by meeting at least one of these criteria: physician diagnosis, use of glucose-lowering drugs or hemoglobin A1c (HbA1c) ≥ 6.5%. History of Cardiovascular Disease (CVD) was identified through self-reports of heart failure, coronary heart disease, stroke, myocardial infarction, or angina. Information on four types of medications used by participants ‒ anti-hypertensive drugs, glucose-lowering drugs, lipid-lowering drugs, and diuretics ‒ was extracted using NHANES prescription drug codes.

### Statistical analysis

Baseline characteristics were compared across BUN/HDL ratio tertiles using weighted Chi-Square tests for categorical variables and weighted analysis of variance (ANOVA) for continuous variables. Three progressively adjusted models were constructed: Model 1 (unadjusted), Model 2 (adjusted for demographic factors), Model 3 (further adjusted for lifestyle factors, dietary protein intake, medical history, and key laboratory parameters) and Model 4, which additionally incorporated fasting metabolic markers (TG and HOMA-IR) in the subset of participants with available data. Subgroup analyses were performed to assess the consistency of associations across predefined population strata. The predictive performance of the BUN/HDL ratio as a standalone variable was first evaluated by calculating the Area Under the Receiver Cperating Characteristic Curve (AUC-ROC) for 5-year mortality, with the optimal clinical cut-point determined by maximizing Youden's index. Predictor variables were selected from the entire dataset using a two-stage approach (Boruta algorithm followed by Adaptive Best Subset Selection, ABESS). The resulting models exhibited an Event Per Variable (EPV) ratio for the multivariable models was approximately 128.6 for all-cause mortality in Model 2, 59.7 in Model 3, 47.4 for cardiovascular mortality in Model 2, and 20.5 in Model 3. The prediction models had an EPV of approximately 128.6 for all-cause mortality and 81.3 for cardiovascular mortality. They were well above 10, which supports model stability. Multicollinearity among selected variables was assessed using Variance Inflation Factor (VIF), with values < 5 indicating acceptable collinearity levels. Model performance (discrimination and calibration) was internally validated using bootstrap resampling (n = 1000 replicates) to obtain bias-corrected confidence intervals. Discrimination was assessed by the AUC-ROC, and calibration by Brier scores and calibration plots. The incremental value of the BUN/HDL ratio was assessed using changes in the AUC, Net Reclassification Improvement (NRI), and Integrated Discrimination Improvement (IDI) and through rigorous nested model comparisons, including Likelihood Ratio (LR) tests and the comparison of Akaike Information Criterion (AIC) values, comparing it to traditional biomarkers and evaluating its addition to the established risk stratification tool. All statistical analyses were conducted using R software (version 4.4.1), with two-sided p-values < 0.05 considered statistically significant.

## Results

### Baseline characteristics

Table 1 presented baseline characteristics stratified by BUN/HDL ratio tertiles among 7,432 participants. Significant differences emerged across groups (p < 0.001). The highest BUN/HDL ratio tertile had significantly higher ages, higher proportions of smoking and lower educational attainment. This group exhibited significantly elevated prevalence of HTN, DM, and CVD, underscoring advanced cardiovascular and metabolic burden. This group also exhibited higher prevalence of CKD stages G3‒G5 and reduced eGFR, suggesting advanced renal impairment. The highest tertile exhibited higher BMI and elevated WBC, TG, HOMA-IR signaling metabolic disorder and systemic inflammation. The prominent reductions in Hb, Alb, and PLT reflected malnutrition and hematologic abnormalities. Medication use patterns rose across tertiles, aligning with greater cardiovascular burden and current guidelines.

**Table 1:** Baseline characteristics of participants according to BUN/HDL ratio tertile groups.

Variables		BUN/HDL			
		T1 (n = 2596)	T2 (n = 2350)	T3 (n = 2486)	p-value
		3.05 (0.02)	4.77 (0.01)	8.19 (0.08)	<0.001
Age	Years	59.56 (0.42)	60.56 (0.37)	63.95 (0.43)	<0.001
Gender					<0.001
	Female	752 (10.5%)	586 (8.1%)	660 (8.2%)	
	Male	1844 (23.0%)	1764 (25.5%)	1826 (24.8%)	
Race					<0.001
	Mexican American	137 (0.9%)	174 (1.1%)	246 (1.4%)	
	Non-Hispanic Black	1062 (7.3%)	593 (3.9%)	418 (2.6%)	
	Non-Hispanic White	1089 (22.7%)	1201 (25.5%)	1420 (26.1%)	
	Other Hispanic	130 (0.8%)	170 (1.1%)	205 (1.3%)	
	Other/Multi-Racial	178 (1.8%)	212 (1.9%)	197 (1.7%)	
Marital status					<0.001
	Married/Cohabiting	1451 (20.2%)	1507 (23.6%)	1477 (21.4%)	
	Other marital status	1145 (13.2%)	843 (10.0%)	1009 (11.6%)	
PIR					0.046
	Not poor	2144 (29.6%)	1971 (30.5%)	2033 (29.4%)	
	Poor (PIR<1)	452 (3.8%)	379 (3.1%)	453 (3.6%)	
Education level					<0.001
	Less than high School	213 (1.5%)	247 (1.7%)	360 (2.4%)	
	High school graduate	380 (3.7%)	320 (3.2%)	341 (3.4%)	
	Some college or higher	2003 (28.2%)	1783 (28.7%)	1785 (27.2%)	
Smoking status					<0.001
	Never	1238 (16.9%)	1161 (18.1%)	1120 (16.0%)	
	Before	823 (10.5%)	847 (11.4%)	1073 (13.4%)	
	Current	533 (6.0%)	342 (4.0%)	293 (3.6%)	
Drinking status					<0.001
	No	1918 (26.1%)	1910 (28.9%)	2123 (30.6%)	
	Yes	409 (6.8%)	236 (4.8%)	162 (2.8%)	
Dietary protein intake	g/day	79.33 (0.96)	86.00 (1.13)	85.48 (1.27)	0.012
Exercise level					0.242
	Sedentary	1595 (19.3%)	1426 (18.2%)	1643 (19.5%)	
	Low activity	502 (7.1%)	481 (7.8%)	472 (7.1%)	
	Moderate	104 (1.3%)	105 (1.5%)	75 (1.3%)	
	High activity	395 (5.8%)	338 (6.0%)	296 (5.1%)	
HTN					<0.001
	No	1026 (15.2%)	826 (14.1%)	620 (10.1%)	
	Yes	1570 (18.2%)	1524 (19.4%)	1866 (23.0%)	
DM					<0.001
	No	2147 (29.8%)	1739 (27.7%)	1403 (21.4%)	
	Yes	387 (3.6%)	557 (5.9%)	1021 (11.7%)	
Hyperlipidemia					<0.001
	No	1351 (16.5%)	1148 (15.7%)	831 (10.1%)	
	Yes	1245 (16.8%)	1202 (17.8%)	1655 (23.0%)	
CVD					<0.001
	No	2158 (28.8%)	1800 (27.2%)	1588 (23.2%)	
	Yes	430 (4.7%)	538 (6.4%)	878 (9.7%)	
CKD stage					<0.001
	G2	2430 (31.7%)	2018 (30.1%)	1439 (22.5%)	
	G3	160 (1.6%)	320 (3.2%)	873 (9.1%)	
	G4 or G5	6 (0.1%)	12 (0.1%)	174 (1.5%)	
BMI		27.81 (0.18)	29.53 (0.16)	30.88 (0.16)	<0.001
eGFR	mL/min/1.73m^2^	78.76 (0.26)	75.97 (0.28)	66.33 (0.54)	<0.001
WBC	×10⁹/L	6 .83 (0.05)	7.15 (0.06)	7.79 (0.12)	<0.001
Hb	g/dL	14.48 (0.05)	14.51 (0.04)	14.11 (0.06)	<0.001
PLT	×10⁹/L	2 30.43 (1.36)	224.09 (1.60)	223.84 (1.75)	<0.001
Alb	g/L	42.62 (0.11)	42.42 (0.09)	41.71 (0.10)	<0.001
TC	mmol/L	5.16 (0.04)	4.94 (0.03)	4.66 (0.04)	<0.001
TG	mmol/L	1.16 (0.03)	1.46 (0.0.03)	1.88 (0.06)	<0.001
HOMA-IR		2.88 (0.09)	4.05 (0.14)	6.51 (0.46)	<0.001
HDL	mmol/L	1.61 (0.01)	1.28 (0.01)	1.05 (0.01)	<0.001
BUN	mmol/L	4.81 (0.04)	6.05 (0.04)	8.44 (0.09)	<0.001
Cr	mg/dL	1.10 (0.01)	1.13 (0.00)	1.39 (0.02)	<0.001
UA	mg/dL	350.45 (1.95)	371.41 (2.24)	393.85 (2.24)	<0.001
Serum sodium	mmol/L	139.54 (0.10)	139.75 (0.11)	139.46 (0.12)	<0.001
Antihypertensive					<0.001
	No	1746 (24.0%)	1382 (21.5%)	1097 (16.0%)	
	Yes	850 (9.5%)	968 (12.0%)	1389 (17.0%)	
Glucose lowering					<0.001
	No	2350 (31.1%)	1978 (29.4%)	1750 (24.5%)	
	Yes	246 (2.3%)	372 (4.1%)	736 (8.5%)	
Lipid lowering					<0.001
	No	2166 (27.8%)	1784 (25.7%)	1706 (22.8%)	
	Yes	430 (5.6%)	566 (7.8%)	780 (10.2%)	
Diuretic					<0.001
	No	2192 (29.2%)	1909 (28.1%)	1674 (23.9%)	
	Yes	404 (4.2%)	441 (5.4%)	812 (9.1%)	

All values are presented as number (n) and proportion (%) for categorical variables, assessed via weighted chi-square tests, or mean (standard error) for continuous variables, assessed via weighted analysis of variance (ANOVA). T1, Tertile 1; T2, Tertile 2; T3, Tertile 3; PIR, Ratio of family Income to poverty; HTN, Hypertension; DM, Diabetes Mellitus; CVD, Cardiovascular Disease; BMI, Body Mass Index; eGFR, Estimated Glomerular Filtration Rate; WBC, White Blood Cell count; Hb, Hemoglobin; PLT, Platelet count; Alb, Albumin; TC, Total Cholesterol; UA, Uric Acid; TG, Triglycerides; HOMA-IR, Homeostatic Model Assessment of Insulin Resistance; HDL, High-Density Lipoprotein cholesterol; BUN, Blood Urea Nitrogen; Cr, Creatinine.

### Association of BUN/HDL with all-cause and cardiovascular mortality

During a median follow-up of 71-months (5.9-years), there were 1671 (23.8%) all-cause deaths and 575 (8.2%) cardiovascular deaths among 7432 participants. Elevated BUN/HDL levels demonstrated a significant association with increased all-cause mortality and cardiovascular mortality (Table 2a, Table 2b). In the unadjusted model (Model 1), the Hazard Ratio (HR) was 1.135 (95% CI: 1.118–1.153; p < 0.001) for all-cause mortality and 1.147 (95% CI: 1.125–1.168; p < 0.001) for cardiovascular mortality. After adjusting for demographic factors including age, gender, race, education level, PIR and marital status (Model 2), the HR was 1.105 (95% CI: 1.086–1.125; p < 0.001) for all-cause mortality and 1.117 (95% CI: 1.094–1.142; p < 0.001) for cardiovascular mortality. With further adjustment for lifestyle factors including smoking, drinking status, exercise level, BMI, medical history including HTN, DM, CVD, hyperlipidemia, and laboratory parameters including WBC, eGFR, Cr, UA, Alb, dietary protein intake and serum sodium in Model 3, the HR was 1.050 (95% CI: 1.021–1.081; p < 0.001) for all-cause mortality and 1.062 (95% CI: 1.020–1.105; p = 0.004) for cardiovascular mortality. Model 4 further adjusted for fasting triglycerides and HOMA-IR in the sample with available data, showed that the associations were attenuated but remained significant for all-cause mortality (HR = 1.062; 95% CI: 1.024–1.102; p = 0.001) and cardiovascular mortality (HR = 1.066; 95% CI: 1.011–1.125; p = 0.019). Analysis by tertiles revealed consistent findings: the highest Tertile (T3) was associated with elevated risk of all-cause and cardiovascular mortality after full adjustment. In Model 3, compared to T1, T3 individuals had a 27.0% higher risk of all-cause mortality (HR = 1.270, 95% CI: 1.030–1.570; p = 0.027) and a 52.0% higher risk of cardiovascular mortality (HR = 1.520, 95% CI: 1.030‒2.230; p = 0.036). In Model 4, compared to T1, T3 remained associated with a significantly increased risk of all-cause mortality (HR = 1.425, 95% CI: 1.072‒1.895; p = 0.015), while the association with cardiovascular mortality showed non-significant with increased risk (HR = 1.288, 95% CI: 0.800‒2.074; p = 0.298).

**Table 2a:** Cox regression analysis of the association between BUN/HDL ratio and all-cause mortality in patients with renal insufficiency.

BUN/HDL	Model 1		Model 2		Model 3		Model 4	
	HR (95% CI)	p-value	HR (95% CI)	p-value	HR (95% CI)	p-value	HR (95% CI)	p-value
Continuous	1.135 (1.118‒1.153)	<0.001	1.105 (1.086‒1.125)	<0.001	1.050 (1.021‒1.081)	<0.001	1.062 (1.024‒1.102)	0.001
T1	Reference	‒	‒	‒	‒	‒	‒	‒
T2	1.133 (0.954‒1.345)	0.156	1.087 (0.919‒1.285)	0.333	0.930 (0.770‒1.110)	0.406	0.987 (0.742‒1.314)	0.929
T3	2.385 (1.992‒2.856)	<0.001	1.897 (1.584‒2.272)	<0.001	1.270 (1.030‒1.570)	0.027	1.425 (1.072‒1.895)	0.015

**Table 2b:** Cox Regression analysis of the association between BUN/HDL ratio and cardiovascular mortality in patients with renal insufficiency.

BUN/HDL	Model 1		Model 2		Model 3		Model 4	
	HR (95% CI)	p-value	HR (95% CI)	p-value	HR (95% CI)	p-value	HR (95% CI)	p-value
Continuous	1.147 (1.125‒1.168)	<0.001	1.117 (1.094, -1.142)	<0.001	1.062 (1.020‒1.105)	0.004	1.066 (1.011‒1.125)	0.019
T1	Reference	‒	‒	‒	‒	‒	‒	‒
T2	1.342 (1.007‒1.787)	0.044	1.290 (0.970‒1.715)	0.080	1.050 (0.740‒1.480)	0.797	0.998 (0.621‒1.604)	0.995
T3	3.039 (2.320‒3.980)	<0.001	2.381 (1.790‒3.167)	<0.001	1.520 (1.030‒2.230)	0.036	1.288 (0.800‒2.074)	0.298

Model 1 was not adjusted for any covariate.

Model 2 was adjusted for age, gender, race, education, PIR, marital status.

Model 3 was adjusted for age, gender, race, education, PIR, marital status, smoking status, drinking status, exercise level, BMI, hypertension, diabetes mellitus, cardiovascular disease, hyperlipidemia, eGFR, Cr, WBC, UA, Alb, dietary protein intake,serum sodium.

Model 4 was adjusted for age, gender, race, education, PIR, marital status, smoking status, drinking status, exercise level, BMI, hypertension, diabetes mellitus, cardiovascular disease, hyperlipidemia, eGFR, Cr, WBC, UA, Alb, dietary protein intake, serum sodium, TG, HOMA-IR.

### Subgroup analysis

Subgroup analyses were conducted to evaluate the association between BUN/HDL ratio and both mortality outcomes across various subgroups (Table 3a, Table 3b). BUN/HDL levels maintained significant associations with both all-cause and cardiovascular mortality in most subgroups.

**Table 3a:** Subgroup analysis of the association between BUN/HDL ratio and all-cause mortality in patients with renal insufficiency.

Subgroup			HR (95% CI)	p trend	p interaction
Age					0.025
	≥ 65y	n=4103	1.062 (1.030‒1.094)	<0.001	
	< 65y	n=3329	1.009 (0.941‒1.044)	0.732	
Gender					0.052
	Male	n=5434	1.051 (1.012‒1.090)	0.009	
	Female	n=1998	1.065 (1.030‒1.101)	<0.001	
HTN					0.016
	Yes	n=4960	1.035 (1.003‒1.070)	0.032	
	No	n=2472	1.127 (1.052‒1.207)	<0.001	
DM					0.159
	Yes	n=5289	1.037 (0.990‒1.087)	0.124	
	No	n=1965	1.073 (1.037‒1.111)	<0.001	
Hyperlipidemia					0.131
	Yes	n=4102	1.035 (1.003‒1.067)	0.031	
	No	n=3330	1.085 (1.028‒1.145)	0.003	
CKD stage					0.002
	G2	n=5887	1.079 (1.030‒1.131)	0.002	
	G3	n=1353	1.058 (1.007‒1.111)	0.025	
	G4 or G5	n=192	0.956 (0.888‒1.029)	0.228

**Table 3b:** Subgroup analysis of the association between BUN/HDL ratio and cardiovascular mortality in patients with renal insufficiency.

Subgroup			HR (95% CI)	p trend	p interaction
Age					0.003
	≥ 65y	N=4103	1.064 (1.019‒1.110)	0.005	
	< 65y	N=3329	1.037 (0.947‒1.137)	0.430	
Gender					0.766
	Male	N=5434	1.078 (1.020‒1.139)	0.008	
	Female	N=1998	1.060 (1.016‒1.106)	0.007	
HTN					0.004
	Yes	N=4960	1.043 (0.997‒1.093)	0.070	
	No	N=2472	1.187 (1.057‒1.332)	0.004	
DM					0.646
	Yes	N=5289	1.073 (1.006‒1.144)	0.031	
	No	N=1965	1.072 (1.016‒1.132)	0.012	
Hyperlipidemia					0.099
	Yes	N=4102	1.055 (1.005‒1.107)	0.030	
	No	N=3330	1.110 (1.000‒1.232)	0.049	
CKD stage					0.040
	G2	N=5887	1.127 (1.050‒1.209)	<0.001	
	G3	N=1353	1.066 (0.990‒1.148)	0.089	
	G4 or G5	N=192	0.942 (0.810‒1.094)	0.432	

After Bonferroni adjustment, significant interactions persisted for CKD stage in predicting all-cause mortality and for age and hypertension in predicting cardiovascular mortality.

BUN/HDL showed significant predictive value in individuals aged ≥ 65-years for both all-cause (HR = 1.062, 95% CI: 1.030‒1.094; p < 0.001) and cardiovascular mortality (HR = 1.064, 95% CI: 1.019‒1.110; p = 0.005). The BUN/HDL association persisted in both sexes for all-cause (males: HR = 1.051, p = 0.009; females: HR = 1.065, p < 0.001) and cardiovascular mortality (males: HR = 1.078, p = 0.008; females: HR = 1.060, p = 0.007). For cardiovascular mortality, the hypertension interaction (p = 0.004) revealed a more prominent association in non-hypertensive individuals (HR = 1.187, 95% CI: 1.057‒1.332; p = 0.004) compared to those with hypertension (HR=1.043, 95% CI: 0.997‒1.093; p = 0.070). BUN/HDL exhibited differential predictive value across renal function strata, with the strongest signals in G2 (HR = 1.127, 95% CI: 1.050–1.209; p < 0.001) stage.

This pattern suggested that BUN/HDL ratio may serve as an early marker for cardiovascular and overall mortality risk among mildly reduced eGFR individuals.

### Univariate predictive performance of the BUN/HDL

**Fig. 2:**
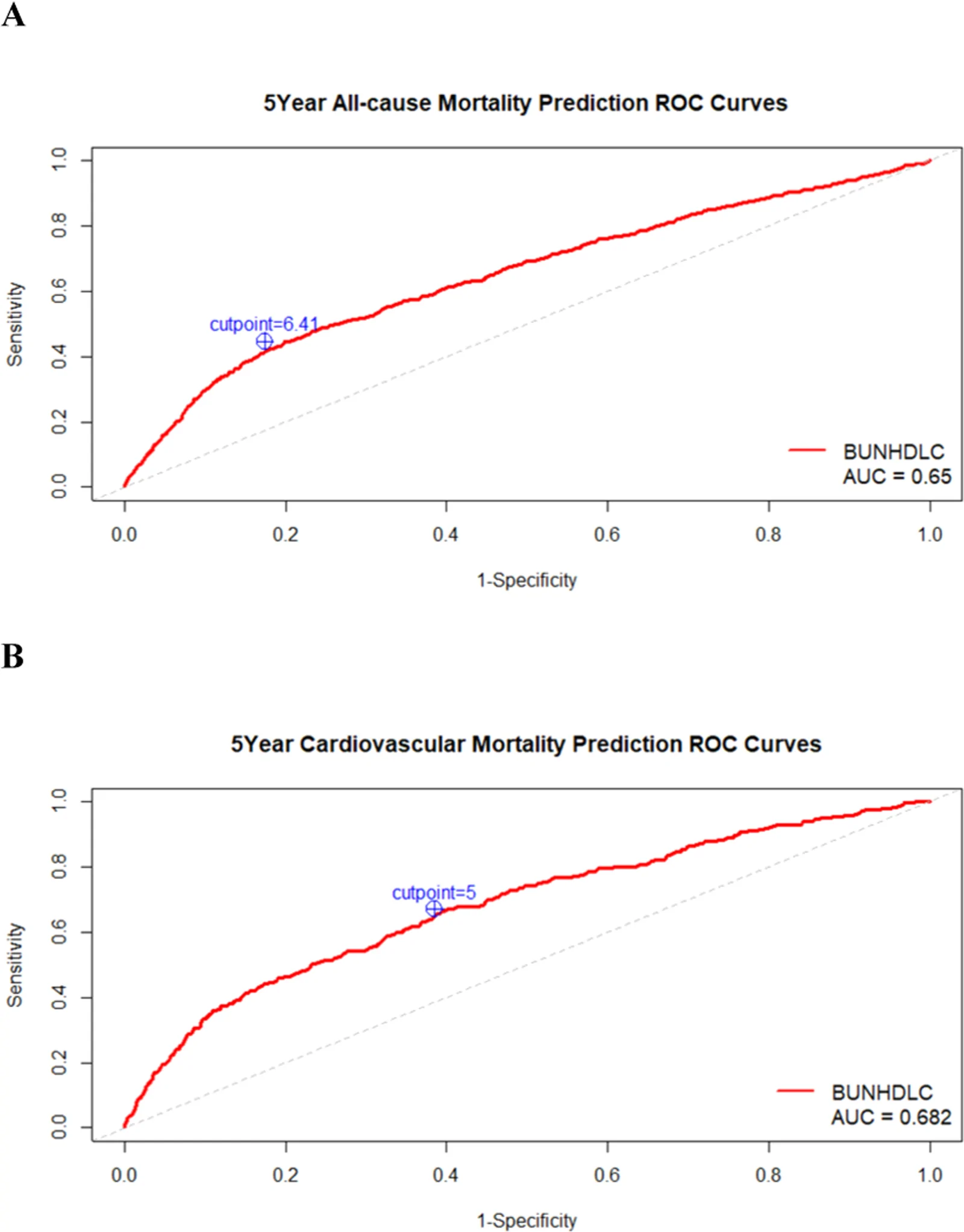
**(A and B) Univariate ROC curve of the BUN/HDL ratio for 5-year mortality prediction.** (A) Receiver Operating Characteristic (ROC) curve for 5-year all-cause mortality. The area under the curve (AUC) is 0.65, with an optimal cut-point of 6.41 determined by Youden's index. (B) ROC curve for Cardiovascular Disease (CVD) mortality. The AUC is 0.682, with an optimal cut-point of 5.00.

The univariate discriminatory ability of the BUN/HDL ratio for predicting 5-year mortality is shown in Figure 2. For all-cause mortality (Fig. 2A), the BUN/HDL ratio yielded an area under the ROC curve (AUC) of 0.65. The optimal cut-point, identified using Youden's index, was 6.41 (sensitivity: 44.7%; specificity: 61.4%). For cardiovascular mortality (Fig. 2B), the BUN/HDL ratio yielded an AUC of 0.682, with an optimal cut-point of 5.00 identified (sensitivity: 67.1%; specificity: 61.4%). The BUN/HDL ratio alone demonstrated poor-to-modest discriminative ability (AUC < 0.70) and is insufficient for clinical use as a standalone test.

### Development of predictive models

The study implemented a two-stage feature selection framework integrating Boruta and ABESS to construct the predictive models for clinical mortality risk stratification. This approach robustly retained the BUN/HDL ratio in both all-cause and cardiovascular mortality models.

For all-cause mortality prediction, the Boruta + ABESS algorithm included 12 key variables: BUN/HDL, age, marital, smoking status, exercise level, BMI, DM, CVD, WBC, eGFR, Hb and Alb. For cardiovascular mortality prediction, 6 critical variables were identified: BUN/HDL, age, CVD, Hb, Alb and diuretic. The BUN/HDL ratio was consistently identified as a core predictor for both all-cause mortality and cardiovascular mortality outcomes. All selected variables demonstrated Variance Inflation Factor (VIF) values within acceptable limits (< 5), suggesting minimal multicollinearity. Formal model comparison assessed the incremental value of the BUN/HDL ratio. For all-cause mortality, adding BUN/HDL to the base model yielded a ΔAUC of 0.003 (p = 0.037); for cardiovascular mortality, the ΔAUC was 0.008 (p = 0.010). Thus, while BUN/HDL is confirmed as a robust predictor within this dataset, it contributes minimally to discriminative ability beyond established risk factors.

### Validation of predictive models

**Fig. 3:**
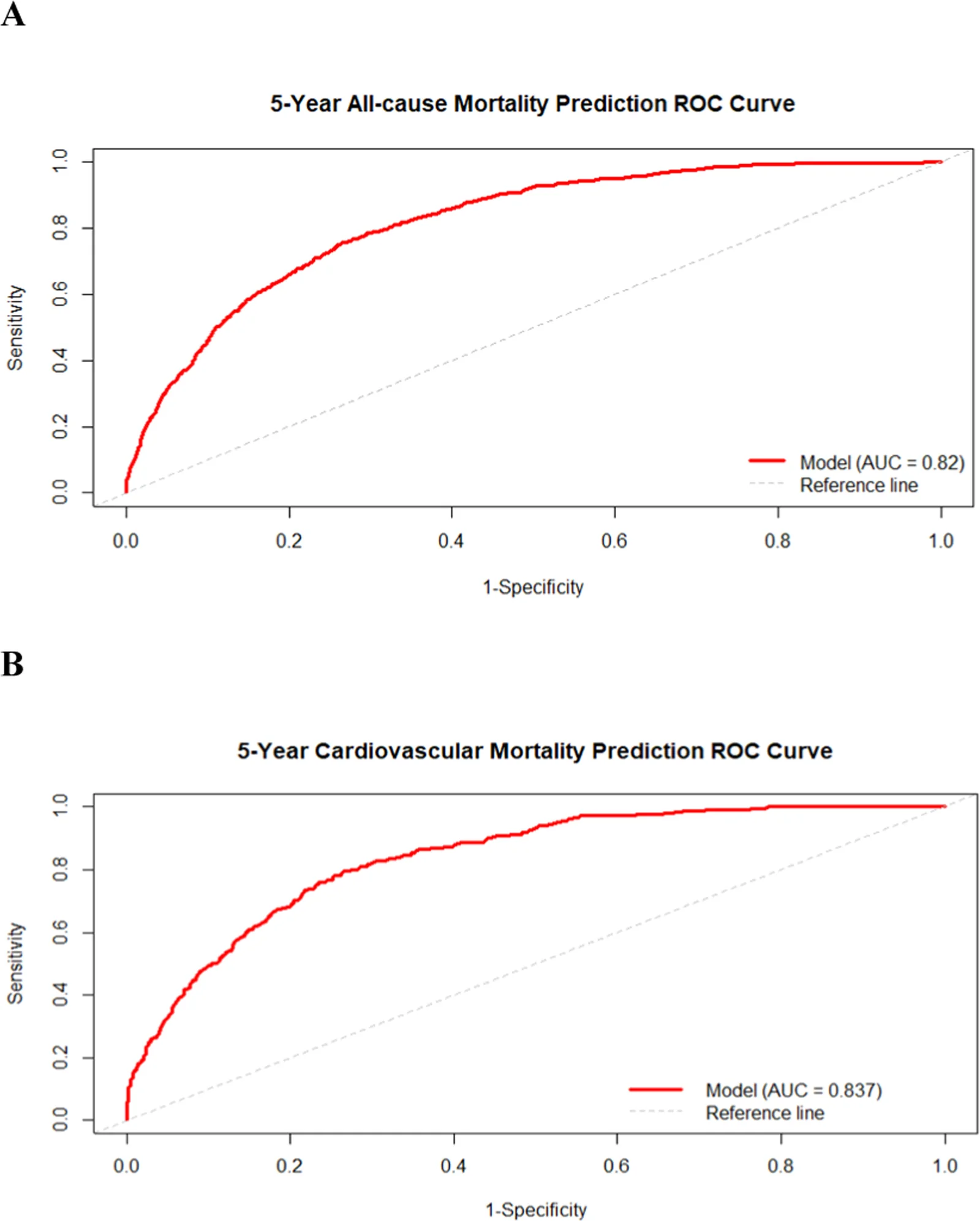
**(A and B) ROC Curves for 5-year all-cause and cardiovascular mortality prediction.** Receiver operating characteristic curves for 5-year mortality prediction. The model for all-cause mortality (A) achieved a bootstrap-validated AUC of 0.820 (95% CI: 0.805–0.834), while the model for cardiovascular mortality (B) achieved an AUC of 0.837 (95% CI: 0.819‒0.858), indicating excellent discriminative ability.

The predictive performance of the multivariable models was rigorously evaluated. Internal validation via bootstrap resampling (n = 1,000 replicates) demonstrated excellent and stable discriminative ability. For 5-year all-cause mortality (Fig. 3A), the model achieved a mean Area Under the Curve (AUC) of 0.820 (95% CI: 0.805‒0.834). For cardiovascular mortality (Fig. 3B), the model achieved a mean AUC of 0.837 (95% CI: 0.819‒0.858). The low coefficients of variation (0.9% and 1.2%, respectively) indicated exceptional stability across bootstrap samples.

**Fig. 4:**
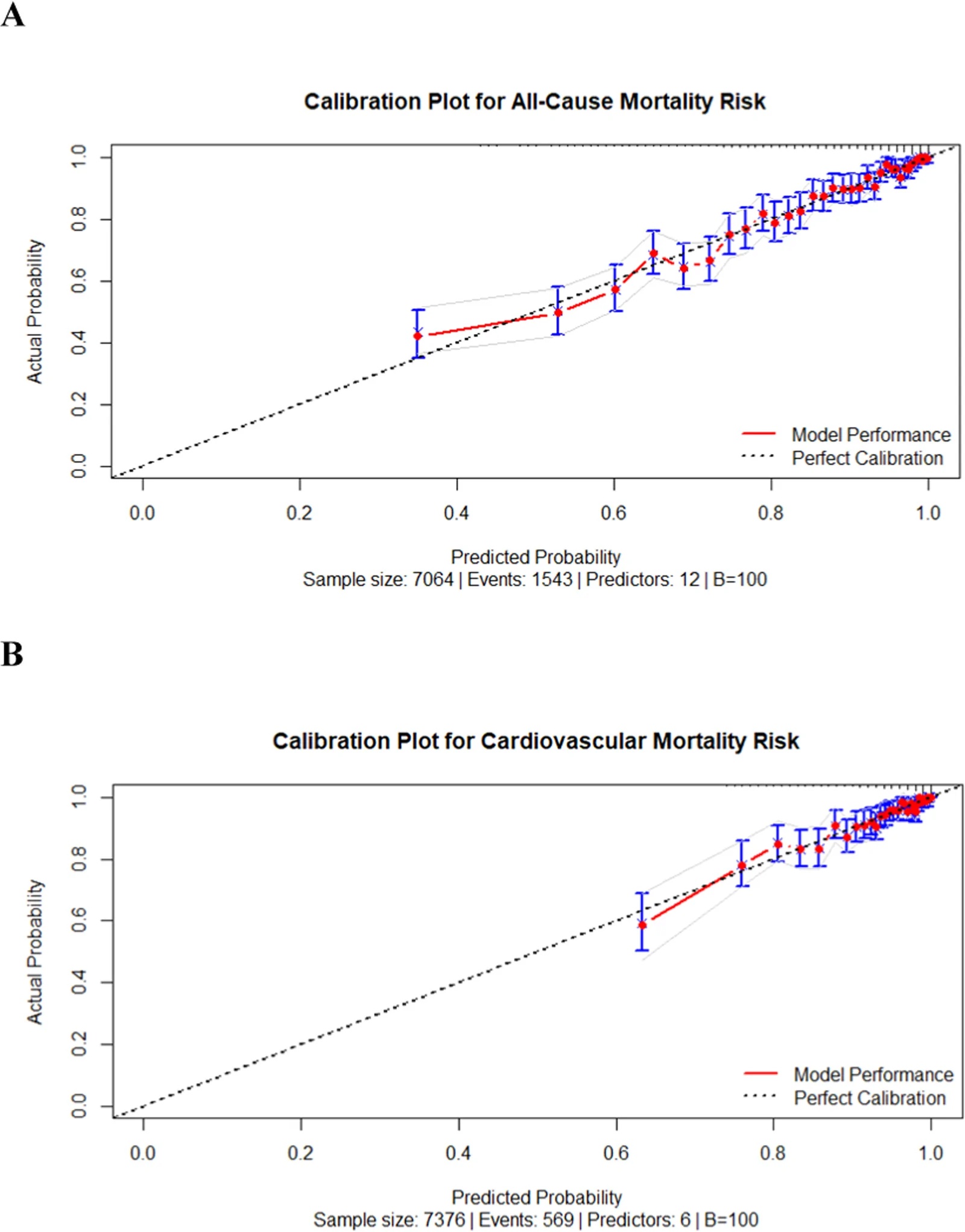
**(A and B) Calibration plots for all-cause and cardiovascular mortality risk.** Calibration plots for 5-year mortality risk prediction. The all-cause mortality model (A) demonstrated precise calibration (Brier score = 0.10; calibration slope = 0.986). The cardiovascular mortality model (B) showed similar accuracy (Brier score = 0.06; calibration slope = 1.030), with both models closely aligned to the ideal line.

Calibration plots confirmed high prediction accuracy across risk strata. For all-cause mortality, the Brier score was 0.10 with excellent alignment to the ideal line (slope = 0.986, 95% CI: 0.930‒1.042; Fig. 4A). For cardiovascular mortality, the model maintained precise calibration with Brier score 0.06 (slope = 1.030, 95% CI: 0.957‒1.103; Fig. 4B). The consistent performance across datasets (AUC differences < 0.02, calibration slope near 1.0) supported clinical applicability.

### Incremental value of the BUN/HDL ratio

The incremental predictive value of the BUN/HDL ratio was assessed in comparison to traditional biomarkers and when added to an established risk stratification tool (Tables 4a and 4b). BUN/HDL ratio demonstrated significant incremental value over several traditional biomarkers. Specifically, it provided a substantial improvement in predicting both all-cause and cardiovascular mortality compared to creatinine (ΔAUC = 0.083 and 0.084, respectively; both p < 0.001) and total cholesterol (ΔAUC = 0.060 and 0.080, respectively; both p < 0.001), supported by significant Net Reclassification Improvement (NRI) and Integrated Discrimination Improvement (IDI) indices (all p < 0.001). Collectively, these findings underscore the incremental prognostic utility of the BUN/HDL ratio beyond traditional biomarkers. The addition of the BUN/HDL ratio to the Framingham risk score yielded a statistically significant improvement in predictive performance for both all-cause (ΔAUC = 0.032, NRI = 0.121) and cardiovascular mortality (ΔAUC = 0.046, NRI = 0.150), with all indices significant (p < 0.001). The improvement in discrimination (ΔAUC < 0.05) is unlikely to be clinically meaningful, suggesting that the BUN/HDL ratio may not warrant supplementation of the Framingham risk score without externally validated net benefit. Complementary decision curve analysis supported this interpretation, with net benefit curves for both models showing substantial overlap across relevant thresholds. (Supplementary Fig. 1 and 2). This integrated predictive value is further substantiated by formal model comparisons (Table 4c). The BUN/HDL ratio consistently achieved the lowest Akaike Information Criterion (AIC) for both outcomes, outperforming models containing its individual components (BUN or HDL alone) or an alternative ratio (Cr/HDL). Notably, the ratio model demonstrated an equivalent fit to the additive model including BUN and HDL as separate covariates. Furthermore, directed Likelihood Ratio (LR) tests indicated that the ratio model did not significantly improve upon the additive model of its individual components for either all-cause (p = 0.625) or cardiovascular mortality (p = 0.288).

**Table 4a:** Incremental value of BUNHDL compared to traditional biomarkers for 5-year mortality prediction.

Traditional Biomarker	Outcome	ΔAUC	p-value (DeLong)	NRI (95% CI)	IDI (95% CI)	p-value (NRI/IDI)
eGFR	All-cause Mortality	-0.010	0.343	-0.020 (-0.045‒ -0.011)	-0.010 (-0.022‒0.002)	0.174
eGFR	Cardiovascular Mortality	-0.012	0.465	-0.031 (-0.074‒0.046)	0.003 (-0.013‒0.018)	0.282
Cr	All-cause Mortality	0.083	<0.001	0.047 (0.032‒0.070)	0.038 (0.028‒0.048)	<0.001
Cr	Cardiovascular Mortality	0.084	<0.001	0.090 (0.064‒0.144)	0.038 (0.024‒0.052)	<0.001
TC	All-cause Mortality	0.060	<0.001	0.046 (0.036‒0.074)	0.039 (0.029‒0.048)	<0.001
TC	Cardiovascular Mortality	0.080	<0.001	0.130 (0.104‒0.233)	0.036 (0.022‒0.049)	<0.001
ALB	All-cause Mortality	-0.021	0.130	-0.060 (-0.098‒ -0.017)	-0.017 (-0.030‒ -0.004)	0.004
ALB	Cardiovascular Mortality	0.034	0.286	-0.008 (-0.047‒0.097)	0.011 (-0.004‒0.027)	0.828

**Table 4b:** Incremental value of adding BUNHDL to traditional risk stratification for 5-year mortality prediction.

Traditional risk stratification	Outcome	ΔAUC	p-value (DeLong)	NRI (95% CI)	IDI (95% CI)	p-value (NRI/IDI)
Framingham	All-cause Mortality	0.032	<0.001	0.121 (0.072‒0.141)	0.032 (0.020‒0.044)	<0.001
Framingham	Cardiovascular Mortality	0.046	<0.001	0.150 (0.106‒0.238)	0.034 (0.019‒0.049)	<0.001

**Table 4c:** Model fit statistics (AIC and LR Tests) for alternative predictors of mortality.

Model	All-cause Mortality			Cardiovascular Mortality		
	AIC	ΔAIC	LR Test vs. Base	AIC	ΔAIC	LR Test vs. Base
Base model	3564.032	17.870	Ref	1907.413	35.717	Ref
+BUN alone	3548.067	1.905	χ² = 17.964, p < 0.001	1875.115	3.419	χ² = 34.298, p < 0.001
+ HDL alone	3564.477	18.315	χ² = 1.555, p = 0.212	1903.472	31.776	χ² = 5.9411, p = 0.015
+ BUN + HDL	3547.923	1.761	χ² = 20.108, p < 0.001	1872.566	0.870	χ² = 38.847, p < 0.001
+ BUN/HDL ratio	3546.162	0.000*	χ² = 9.869, p < 0.001	1871.696	0.000*	χ² = 37.717, p < 0.001
+ Cr/HDL ratio	3564.553	18.391	χ² = 1.4789, p = 0.224	1896.793	25.097	χ² = 12.62, p < 0.001

The base model comprised established risk factors (excluding BUN/HDL): age, marital status, smoking status, exercise level, BMI, DM, CVD, WBC, eGFR, Hb, and Alb for all-cause mortality; and age, CVD, Hb, Alb, and diuretic use for cardiovascular mortality.

## Discussion

This study represented the first large-scale investigation to demonstrate that elevated BUN/HDL ratio was significantly associated with increased risks of all-cause and cardiovascular mortality in patients with mildly reduced eGFR (eGFR < 90 mL/min/1.73 m^2^). Through the Boruta-ABESS algorithm, the BUN/HDL ratio retained as a robust prognostic predictor within the NHANES dataset, while contributing minimally to discriminative ability. The predictive models incorporating this ratio demonstrated strong performance, with AUC values of 0.820 for all-cause mortality and 0.837 for cardiovascular mortality, and maintained excellent calibration (calibration slopes near 1.0) following internal validation. While urea-driven carbamylation is hypothesized to partially explain this association, this study did not measure carbamylated HDL or conduct functional assays. Accordingly, this hypothesis serves solely as a potential biological rationale for future investigation and does not constitute mechanistic evidence.

BUN, the terminal metabolite of protein catabolism, exhibits dual clinical significance. Elevated levels reflect impaired renal function and uremic toxin accumulation, while markedly reduced concentrations, especially in dialysis patients, indicate malnutrition and correlate with increased infection risk and muscle loss.^14^ Regarding cardiovascular risk, elevated BUN levels are closely associated with increased cardiovascular mortality risk in patients with renal dysfunction. Elevated urea concentrations directly affect various cell types, including vascular endothelial cells, smooth muscle cells, and renal tubular cells, significantly increasing intracellular Reactive Oxygen Species (ROS) production and disrupting redox balance, thereby triggering oxidative stress cascades.^15^ This process accelerates endothelial progenitor cell senescence, induces smooth muscle cell apoptosis, damages renal tubular cells, and interferes with insulin signal transduction, reducing insulin sensitivity, collectively forming the pathophysiological basis of cardiovascular complications.^16^^,^^17^ Furthermore, urea metabolite cyanate can mediate lipoprotein carbamylation, forming carbamylated LDL with enhanced pro-inflammatory and pro-coagulant properties. These modified lipoproteins are more likely to deposit in vascular walls, damage endothelial cells, and promote foam cell formation, accelerating atherosclerotic plaque progression and increasing its instability.^18^

HDL levels are reduced in patients with renal insufficiency and serve as an indicator of renal function deterioration.^19^ Studies have demonstrated that individuals with HDL levels below 30 mg/dL face a 10%‒20% higher risk of developing or experiencing worsening renal dysfunction compared to those with levels of 40 mg/dL.^20^ Beyond quantitative reduction, HDL particles in patients with renal insufficiency exhibit significant functional impairment. The apolipoprotein composition of HDL particles changes, with decreased levels of the protective structural protein apolipoprotein A-I (apoA-I) and increased incorporation of the acute-phase reactant Serum Amyloid A (SAA). HDL particles display altered lipid composition, characterized by increased triglyceride content and reduced phospholipids, diminishing particle stability.^21^ Furthermore, the activity of Lecithin-Cholesterol Acyltransferase (LCAT), a key enzyme essential for HDL maturation and function, is significantly impaired. This reduction in LCAT activity results in decreased cholesterol ester formation, preventing HDL from properly maturing into functional spherical particles.^22^ The combination of reduced HDL levels and functional impairment creates a synergistic effect that promotes cardiovascular risk progression in patients with renal insufficiency.

Beyond their individual associations with cardiovascular risk, the BUN/HDL ratio is hypothesized to reflect the integrated burden of urea-driven carbamylation and HDL dysfunction.^12^ Elevated BUN promotes a urea-driven process known as carbamylation, a post-translational modification that compromises HDL's structural and biological activity.^23^ This process impairs HDL's atheroprotective integrity by compromising the cholesterol efflux capacity of ApoA-I, inactivating the antioxidant defenses of Paraoxonase 1 (PON-1) and reducing LCAT activity.^24^ The alterations transform HDL into a pro-atherogenic agent. Clinical data link HDL carbamylation to serum urea in CKD cohorts,^25^ while vitro studies show it abrogates HDL's endothelial-protective functions via abolished PON-1 activity and downregulated VEGFR2/SR-BI signaling in vitro.^26^ Future studies quantifying carbamylated HDL or PON-1 activity against this ratio are needed to test this hypothesis.

The association between the BUN/HDL ratio and mortality remained statistically significant after comprehensive multivariable adjustment. In the adjusted model (Model 3), each unit increase in the ratio was associated with a 5.0% higher risk of all-cause mortality (HR = 1.050, 95% CI: 1.021‒1.081; p < 0.001) and a 6.2% higher risk of cardiovascular mortality (HR = 1.062, 95% CI: 1.020‒1.105; p = 0.004). Recognizing that an eGFR < 90 mL/min/1.73 m^2^ threshold may include younger individuals with normal kidney function, the study verified the robustness of the findings via a sensitivity analysis excluding those < 50-years old. Effect estimates were virtually identical (all-cause HR = 1.051, 95% CI 1.021–1.082; cardiovascular HR = 1.062, 95% CI 1.019–1.106), confirming the stability of the observed association.

To assess the clinical relevance of this effect size, we evaluated the hazard increase over the Interquartile Range (IQR) of the ratio in our cohort (3.48 to 6.46, difference = 2.98 units). This corresponds to a 14.9% higher risk of all-cause mortality and a 18.5% higher risk of cardiovascular mortality for an individual at the 75^th^ percentile compared to one at the 25^th^ percentile ‒ a difference that supports the clinical utility of the ratio. The univariable ROC analysis demonstrated poor-to-modest discriminative ability, showing AUCs of 0.65 for all-cause mortality and 0.682 for cardiovascular mortality.

Bonferroni correction was applied to the subgroup interaction tests. Notably, the subgroup findings are strictly exploratory and hypothesis-generating, thus requiring external validation. It revealed that the predictive power of the BUN/HDL ratio was most pronounced in stage G2, weakened in G3, and became non-significant in patients with advanced (G4‒G5) disease. Emerging studies confirm that mildly reduced eGFR, even at subclinical levels, is associated with increased cardiovascular disease risk.^27^ Notably, in a large-scale clinical study conducted in China, mild renal insufficiency has been shown to confer higher attributable risk for major adverse cardiovascular events during hospitalization compared to moderate to severe renal impairment in Acute Coronary Syndrome (ACS) populations,^28^ highlighting the potential value of the BUN/HDL ratio for the early identification of high-risk individuals. The predictive capacity of the ratio in mildly reduced eGFR individuals is supported by the early activation of key pathophysiological pathways:

Reduced eGFR is still significantly associated with inflammatory markers such as C-Reactive Protein (CRP), Interleukin-6 (IL-6), and SAA in participants without overt renal dysfunction (eGFR > 60 mL/min/1.73 m^2^), indicating that inflammatory responses are activated early and contribute to cardiovascular risk.^29^ Vascular dysfunction manifests early in mild renal insufficiency, as clinical studies have demonstrated that middle-aged and older men with mild kidney disease exhibit lower endothelial function and greater aortic stiffness compared with control men with the degree of vascular dysfunction being directly associated with kidney function.^30^ HDL dysfunction has also been validated in early kidney disease, presenting as a phenotype of low HDL cholesterol with insulin resistance, which is closely associated with elevated monocyte counts and progression of atherosclerosis.^31^

The predictive value of the BUN/HDL ratio was not significant in individuals with eGFR < 60 mL/min/1.73 m^2^ and may be explained by the following reasons: Consistent with CANTOS trial data showing that lipid metrics lose predictive power in advanced CKD while inflammation persists, the attenuated efficacy of the BUN/HDL ratio in moderate-to-severe renal impairment may reflect the waning weight of its lipid (HDL) component relative to inflammation-driven risk.^32^ Despite the most severe uremic burden in eGFR < 30 mL/min/1.73 m^2^, the ratio’s prognostic utility is eclipsed by competing ESRD-specific risks, including ventricular remodeling, mineral-bone disorders, and protein-energy wasting.^33^, ^34^, ^35^ Future studies with larger cohorts are needed to validate the ratio's utility across the full CKD spectrum and to further elucidate these potential explanations.

The stronger association of the BUN/HDL ratio with cardiovascular mortality in non-hypertensive individuals underscores that pathophysiological processes active in the pre-clinical phase contribute significantly to cardiovascular risk. This is supported by evidence of early risk activation in the absence of hypertension. For example, renal function mediates the link between blood pressure and subclinical atherosclerosis in non-hypertensive adults, highlighting the kidney's role in early disease development.^36^ Furthermore, markers such as elevated heart rate and serum uric acid are recognized independent risk factors in young, normotensive individuals.^37^^,^^38^ Collectively, an elevated BUN/HDL ratio in this subgroup likely reflects a state of chronic cardiorenal metabolic dysregulation. Although mechanisms like subclinical volume depletion may acutely influence BUN and represent a pathway to decompensation, the ratio as measured here primarily captures steady-state risk. Therefore, this finding highlights the potential of the BUN/HDL ratio to capture the oxidative stress and lipid metabolic risk active in this pre-clinical phase in identifying high-risk, non-hypertensive individuals, warranting heightened vigilance.

The BUN/HDL ratio offered incremental predictive value for cardiovascular mortality among individuals with mildly reduced eGFR beyond creatinine and total cholesterol. While creatinine primarily reflects glomerular filtration rate,^39^ elevated BUN mediates cardiovascular pathology by inducing vascular oxidative stress and endothelial dysfunction; its derivative cyanate also promotes pro-atherogenic lipoprotein carbamylation. HDL reflects systemic anti-inflammatory and antioxidant capacity, and its depression represents a stable risk trait regardless of lipid-lowering therapy,^40^ providing a less confounded metric. Evaluation of AIC values demonstrated that the BUN/HDL ratio provided superior model fit compared to either component alone (BUN or HDL) or an alternative Cr/HDL ratio, while yielding a fit comparable to ‒ and not significantly improved upon ‒ the additive model including BUN and HDL as separate covariates. Moreover, the modest discrimination gain (ΔAUC < 0.05) over the Framingham risk score suggests that this ratio may not warrant replacing the score without externally validated net benefit.

### Strengths and Limitations

This study introduces a novel composite biomarker and, for the first time, demonstrates its significant associations with cardiovascular and all-cause mortality in mildly reduced eGFR populations. This biomarker is easily measurable and cost-effective, enhancing its feasibility for widespread clinical application and routine screening. It can serve as a valuable tool for identifying high-risk individuals before overt clinical manifestations. This study has several limitations that should be acknowledged. First, the large sample size can inflate statistical significance (very small p-values) for minor effects, necessitating cautious interpretation. Secondly, the findings are confined to individuals with mildly reduced eGFR (< 90 mL/min/1.73 m^2^), which may not represent overt renal injury, particularly given that the majority of participants had normal ACR values (< 30 mg/g). Thus, caution is warranted when extrapolating these findings to populations with overt kidney disease. Thirdly, residual confounding remains possible. Metabolic confounders, such as apolipoprotein B, were unavailable (NHANES 2017‒2018 cycle), and other covariates like triglycerides and HOMA-IR could only be adjusted for in a fasting sample. Unmeasured medication adherence represents an additional source of residual confounding. Fourth, the suggested link to urea-induced HDL carbamylation remains speculative, as this study lacked direct measurements of carbamylated HDL or its functional activity. Finally, this study was validated only within the database, and future studies should include validation across different datasets to confirm the robustness of the biomarker.

## Conclusion

In summary, this study provides preliminary evidence within the NHANES database supporting the BUN/HDL ratio as a novel biomarker associated with mid-term cardiovascular and all-cause mortality in patients with mildly reduced eGFR. While the incremental predictive gain is modest in the present dataset, the ratio’s inherent practicality and cost-effectiveness support its exploratory use as a potential screening tool. Future research should prioritize external validation in independent cohorts, more comprehensive adjustment for metabolic and clinical confounders, direct experimental assessment of the postulated carbamylation mechanism.

## Abbreviations

ASCVD, Atherosclerotic cardiovascular disease; ACC/AHA, American College of Cardiology/American Heart Association; ESC, European Society of Cardiology; CKD, Chronic Kidney Disease; eGFR, Estimated Glomerular Filtration Rate; CKM, Cardiovascular-Kidney-Metabolism; RAAS, Renin Angiotensin Aldosterone System; SNS, Sympathetic Nervous System; BUN, Blood Urea Nitrogen; HDL, High-Density Lipoprotein cholesterol; NHANES, National Health and Nutrition Examination Survey; CDC, Centers for Disease Control and Prevention; IRB, Institutional Review Board; PIR, Poverty-to-Income Ratio; BMI, Body Mass Index; Cr, Creatinine; CKD-EPI, Chronic Kidney Disease Epidemiology Collaboration; NDI, National Death Index; WBC, White Blood Cell; Hb, Hemoglobin; PLT, Platelet; Alb, Albumin; TC, Total Cholesterol; HTN, Hypertension; DM, Diabetes Mellitus; HbA1c, Hemoglobin A1c; CVD, Cardiovascular Disease; ANOVA, Weighted Analysis of Variance; ABESS, Adaptive Best Subset Selection; VIF, Variance Inflation Factor; AUC-ROC, Area under the receiver operating characteristic curve; CI, Confidence Interval; HR, Hazard Ratio; ROS, Reactive Oxygen Species; apoA-I, Apolipoprotein A-I; SAA, Serum amyloid A; LCAT, Lecithin-Cholesterol Acyltransferase; ACS, Acute Coronary Syndrome; CRP, C-Reactive Protein; IL-6, Interleukin-6; ESRD, End-Stage Renal Disease; VLDL, Very Low-Density Lipoprotein; ACR, Albumin-to-Creatinine Ratio.

## Ethics approval and consent to participate

The NHANES study protocols were approved by the NCHS Ethics Review Board. Data collection for the 2007‒2010 cycles operated under Protocol #2005-06, and for the 2011‒2016 cycles under Protocol #2011-17. For the 2017‒2018 cycle, Protocol #2011-17 was in effect through October 25, 2017, and Protocol #2018-01 was effective beginning October 26, 2017.

## Consent for publication

Not applicable.

## Data statement

The data used in this study are publicly available from the National Health and Nutrition Examination Survey (NHANES) (https://wwwn.cdc.gov/nchs/nhanes) and its linked mortality database (https://www.cdc.gov/nchs/data-linkage/mortality.htm).

## Authors’ contributions

Zhong Chen designed the study, Shijia Jin and Yilin He performed data management, and data analysis. Shijia Jin and Yifan Zhang wrote the manuscript. Yilin He and Yifan Zhang edited the figures and article format. Yifan Zhang and Zhong Chen made key revisions to the article. All authors contributed to the article and approved the submitted version.

## Funding

None declared.

## Data availability statement

The datasets generated and/or analyzed during the current study are available from the corresponding author upon reasonable request.

## Acknowledgments

None declared.

## Declaration of competing interest

The authors declare no conflicts of interest.

## Appendix Supplementary materials

Image, application 1
