# Supplementary material for: Association of blood urea nitrogen to high-density lipoprotein ratio with all-cause and cardiovascular mortality in individuals with mildly reduced eGFR
Source: Clinics (Sao Paulo). 2026 Sep 5;81:101096. doi: 10.1016/j.clinsp.2026.101096 (PMC13579277; doi:10.1016/j.clinsp.2026.101096)
Supplement: Supplementary file 1 [file mmc1.doc]

CLINICS-D-25-01762_ **Supplementary Material**

**Supplementary Figure 1** Decision curve analysis comparing the Framingham risk score with and without the BUN/HDL ratio for all-cause mortality.

**Supplementary Figure 2** Decision curve analysis comparing the Framingham risk score with and without the BUN/HDL ratio for cardiovascular mortality.
